# Circulating extracellular vesicles as predictive biomarkers of progressive interstitial lung disease in systemic sclerosis—a prospective cohort study

**DOI:** 10.3389/fmed.2025.1594201

**Published:** 2025-04-30

**Authors:** J. Colic, I. Pruner, N. Damjanov, J. Antovic, M. Sefik-Bukilica, M. Matucci Cerinic, A. Antovic

**Affiliations:** ^1^Department of Rheumatology Unit, Faculty of Medicine, Institute of Rheumatology, University of Belgrade, Belgrade, Serbia; ^2^Division of Rheumatology, Department of Medicine Solna, Karolinska Institutet, Stockholm, Sweden; ^3^Department of Molecular Medicine and Surgery, Karolinska Institutet, Stockholm, Sweden; ^4^Unit of Immunology, Rheumatology, Allergy and Rare Diseases, IRCCS San Raffaele Hospital, Milano, Italy; ^5^Department of Rheumatology, Karolinska University Hospital, Stockholm, Sweden

**Keywords:** systemic sclerosis, extracellular vesicles, progressive ILD, pathogenesis, ICAM1

## Abstract

**Objectives:**

To assess in patients with systemic sclerosis (SSc) the concentration of different subpopulations of circulating extracellular vesicles (EVs) and their association with the progression of interstitial lung disease (PF-ILD).

**Methods:**

The prospective study included 59 SSc cases, 54% with interstitial lung disease (ILD). Plasma levels of EVs were analysed with flow cytometry and labelled as endothelial (EEVs), platelet (PEVs), leucocyte (LEVs), and EVs, expressing ICAM1, TF, or HMGB1. The presence of ILD was defined by HRCT. Lung functional tests were done every 3–6 months over a 3-year follow-up period. PF-ILD was defined as ≥10% decline of FVC % from baseline, or ≥5–9% along with a decline in DLCO of ≥15%.

**Results:**

At baseline, 32/59 SSc patients had ILD, with a median disease duration of 3 years, and 38% were therapy naïve. In ILD patients, increased levels of all investigated EVs were found in respect to SSc patients without ILD (*p* < 0.05). Therapy naïve ILD cases had altered only ICAM1 + EVs compared to treated (*p* < 0.05). Multivariate regression analysis (MR) showed an independent association of PEVs (OR 1.004, 95% CI 1.001–1.01) and ICAM1 + EVs (OR 1.3, 95% CI 1.1–1.5) with ILD. During the follow-up period, 12/32 ILD patients developed PF-ILD, and in this group, the levels of all explored EVs were elevated compared to those without PF-ILD (*p* < 0.05). In an ROC analysis, all EVs showed a good ability to identify PF-ILD patients (*p* < 0.05). Cox MR confirmed the independent predictive value of ICAM1 + EVs (HR 1.1, 95% CI 1.01–1.1) with SSc PF-ILD.

**Conclusion:**

Circulating EV levels are increased in SSc and correlate with ILD. In particular, ICAM1 + EVs may be a novel biomarker of PF-ILD, identifying SSc patients at high risk of progression who may require early aggressive treatment. Based on our results, the role of EVs in the pathogenesis and progression of ILD should be investigated further.

## Highlights

- Circulating EVs of different cell origin may play a role in the pathogenesis of ILD in systemic sclerosis.
- EVs expressing ICAM1 may be a novel biomarker of progressive ILD.
- EVs expressing ICAM1 may serve as a biomarker to stratify patients for early aggressive treatment.

## Background

Pulmonary involvement has been seen in over 70% of systemic sclerosis (SSc) patients. Two major manifestations are interstitial lung disease (ILD), which represents the most common cause of SSc-related death, and pulmonary vascular disease (1). Diagnosis of ILD is still challenging since the majority of patients are asymptomatic in the early stage, and there are no biomarkers in clinical use with validated diagnostic and prognostic features (2).

The most frequent methods for ILD assessment are lung function tests (LFTs) and high-resolution computed tomography (HRCT). A decline across two LFTs [measuring forced vital capacity (FVC%) and diffusing capacity of the lungs for carbon monoxide (DLCO%)] is indicative of ILD. Definitive diagnosis is based on HRCT imaging, which represents the gold standard. The most common pathological pattern of ILD is nonspecific interstitial pneumonia, which mirrors the finding of ground glass opacities with peripheral distribution, reticulation, and traction bronchiectasis (3).

Although the pathogenesis of SSc-ILD remains elusive, it has been known that vasculopathy plays a crucial role driven by the interplay between endothelial and alveolar injury, the defective healing process, activation of fibroblasts, and inflammatory response. SSc pathogenesis thus results from cell–cell, cell–cytokine, and cell–matrix interactions (4).

Transforming growth factor beta (TGF-β) is considered a master player in the progression of SSc-ILD. Further, there is a growing body of evidence that some pro-inflammatory markers [e.g., interleukin 6 (IL-6)] (5), along with markers of endothelial activation and injury [intercellular adhesion molecule 1 (ICAM1) and vascular endothelial growth factor (VEGF)] (6, 7), may also have an essential role in SSc-ILD pathogenesis. The role of High-mobility group box-1 protein (HMGB1) in mediating inflammation, oxidative stress, and the endothelial- or epithelial-mesenchymal transition in SSc-related lung disease has been highly debated (8).

Extracellular vesicles (EVs) are membrane-coated vesicles arising from outward budding of the cell membrane in response to different stimuli. EVs behave as critical mediators of intercellular communication by transmitting signals (proteins and nucleic acids) in an autocrine, paracrine, and even endocrine fashion (9). Previous experimental studies have shown possible implications of EVs in all the above-mentioned key processes underlying SSc-ILD pathogenesis (6). By exposing phosphatidyl serine (PS) together with tissue factor (TF) as a consequence of membrane flipping, EVs are involved in the vasculopathy, initiation, and amplification of the inflammatory process and coagulation (6). Although EVs may derive from virtually any cell type, those generated from endothelial cells (EEVs), platelets (PEVs), and leucocytes (LEVs) are comprehensively explored in SSc, with divergent results (8). Recently, a direct profibrotic role of EVs has been shown in lung fibrosis related to SSc (10, 11). To date, only a few studies have reported elevated levels of EVs in SSc patients with lung involvement, mainly EEVs and PEVs (10, 12–14, 48). However, clinical studies exploring the role of EVs in the progression of SSc-ILD have not been performed previously.

Considering the complex pathogenesis of SSc-ILD, we decided to perform a pilot study exploring the levels of circulating EEVs, PEVs, and LEVs, along with EVs exposing TF, ICAM1, or HMGB1 in patients with SSC-ILD, as well as a cohort that investigates the onset of ILD progression over a 3-year follow-up period.

## Methods

### Study design

This was a nested case-control study within a prospective cohort study of 59 patients with SSc followed at the outpatient clinic of the referral Serbian hospital for SSc (the Institute of Rheumatology in Belgrade) between 2017 and 2021. Patients aged >18 years and meeting the 2013 American College of Rheumatology (ACR)/European Alliance of Associations for Rheumatology (EULAR) classification criteria (15) were eligible for inclusion. The exclusion criteria were the following: other autoimmune diseases, including thyroiditis, asthma, and chronic obstructive pulmonary disease; prior cardiovascular events; diabetes mellitus; liver and renal insufficiency; hemostatic disorders; inflammatory bowel diseases; pregnancy; acute infections; and neoplastic diseases. Before inclusion, the dose of all ongoing medicines was stable for at least 6 months, and the last cyclophosphamide (Cyc) infusion was given at least 3 months prior to inclusion. Forty-six healthy, age and sex-matched individuals were also included as controls.

### Clinical assessment and evaluation instruments

All patients underwent a physical examination at baseline. The extent of skin involvement was estimated with the modified Rodnan Skin Score (mRSS) by summing skin thickness measurements determined by palpation on a scale of 0–3 in 17 body areas (16). The presence of telangiectasias, sclerodactyly, contractures, pitting scars, digital ulcers, and contractures was assessed. The following data were collected from the medical records: age, sex, smoking habits, comorbidities, disease duration (time from the first non-Raynaud phenomenon sign), age at disease onset, and ongoing therapy, including the cumulative dose of Cyc and daily dose of glucocorticoids (GC). Patients were classified into diffuse cutaneous (dcSSc) or limited cutaneous SSc (lcSSc) subsets, according to LeRoy et al. (17). Disease duration of less than 3 years in patients with dSSc, or less than 5 years in those with lSSc, was considered to be the early disease stage (18).

ILD was defined by the presence of typical features, such as ground-glass opacities with peripheral distribution, reticulation, and traction bronchiectasis on a high-resolution CT (HRCT) scan of the chest described by an experienced radiologist. In addition, prior to inclusion, the following assessments were done: LFTs (FVC% and DLCO%) for examination of ILD severity (19), Doppler echocardiography to estimate systolic pulmonary artery pressure (sPAP), and nailfold videocapillaroscopy (NVC). According to NVC features, patients were classified as “early,” “active,” and “late” patterns (20). Disease activity was assessed by the EUSTAR activity index, considering active disease with a score ≥2.5 (21). The functional status of patients was evaluated with the Scleroderma Health Assessment Questionnaire-Disability Index. The presence and severity of respiratory symptoms were assessed using the Visual Analogue Scale (VAS) for lung affection (22).

### Longitudinal study

Interstitial lung disease progression (PF-ILD) has been evaluated as a continual change in LFTs, with an FVC% decline from baseline of ≥10% or ≥5–9%, along with a DLCO% decline of ≥15% (5). The occurrence of PF-ILD was checked every 3–6 months over 3 years of follow-up. Along with PF-ILD onset, prevalent ILD cases and escalation of immunosuppressive treatments were also recorded. Patients without ILD at baseline who experienced worsening respiratory symptoms, significant decline in LFTs (5), or X-ray results suggesting ILD during the follow-up period were referred for a HRCT for a definitive assessment of ILD.

### Blood sampling

Participants were sampled only at baseline. Peripheral venous blood was collected, prepared, and transported to Karolinska Institutet, Stockholm, Sweden, for further analysis, as previously described (23).

### Isolation of EVs

Plasma samples were tested for the presence of EVs using flow cytometry according to the previously established method (24). EVs were labelled with lactadherin—FITC (BD Biosciences, United States) to identify PS + EVs, together with particular antibodies for determining specific EVs: CD144 (BD Biosciences, United States, No. 560410) endothelial EVs, CD42b (BD Biosciences, United States, No. 555473)—platelet EVs, CD45 (BD Biosciences, United States, No. 555485)—leucocyte EVs, CD142 (Bio-Techne, United Kingdom, No. FAB23391A)—EVs expressing tissue factor (TF + EVs), CD54 (BD Biosciences, United States, No. 559771)—EVs expressing intercellular adhesion molecule 1 (ICAM1 + EVs) and high mobility group box 1 (Bio-Techne, United Kingdom, No. IC1690A) for EVs expressing HMGB1 (HMGB1 + EVs).

EVs were measured by flow cytometry on a BD FACSCanto^™^ instrument (25), using conjugate isotype-matched immunoglobulins (IgG1-FITC, IgG1-PE, and IgG1-APC) with no reactivity against human antigens and Triton X 100 lysed EVs to define the positive and negative gates. EVs were defined as particles <1.0 μm in size and positive for lactadherin.

### Analysis of serological markers

Antinuclear, anti-centromere (ACA), and anti-topoisomerase I antibodies (ATA), and routine laboratory analyses, including pro-inflammatory marker C-reactive protein (CRP), were carried out for all participants at inclusion, using standard methods, at the Institute of Rheumatology.

The serum concentrations of endothelial injury markers ICAM-1 and VEGF and another marker of inflammation (IL-6) were measured by ELISA (Quantikine R&D Systems).

### Ethical considerations

The local ethics committee of the Institute of Rheumatology approved this study (No. 29/1-110), and all participants signed written informed consent before enrolment.

### Statistical analysis

Descriptive statistics were used to summarise the characteristics of the participants, depending on normality distribution and the features of the variables. The difference across the three groups was tested using either ANOVA with the *post hoc* test, or the Kruskal–Wallis test with a pairwise *post hoc* Bonferroni test. The difference in frequencies was assessed by Pearson’s *χ*^2^ or Fisher exact test. The difference in continual variables between SSc-ILD and non-SSc-ILD groups, or PF-ILD and non-PF-ILD groups, was explored by either the Student’s *t*-test or the Mann–Whitney *U* test, depending on normal distribution. Spearman’s linear correlation analysis was run to examine the strength and direction of the correlation between the investigated EVs and other continuous variables of interest. Univariate logistic regression assessed the strength of the association of all tested laboratory markers with ILD present. Afterwards, multivariate logistic regression models were built to identify independent laboratory risk factors for the presence of ILD. As covariates, continuous laboratory variables were included in models that previously showed a significant association with the presence of ILD and those already identified in the literature. The strength and direction of the association between continuous variables and the presence of ILD were presented as the odds ratio (OR) and 95% confidence intervals (CI). In order to identify serum markers that may independently influence ICAM1 + EVs plasma levels within the SSc-ILD cohort, the multivariate linear regression was subsequently performed using a forward model including covariates, which has previously shown significant correlation with ICAM1 + EVs. A receiver-operating characteristic (ROC) explored the overall discriminatory value of different EVs. To assess the predictive value of EVs in PF-ILD development, a multivariate Cox regression analysis, including serum markers as covariates, along with CRP, as a well-known biomarker of ILD progression (5), was run. The final forward Cox regression model was then built. In addition to laboratory data, the model included continuous variables regarding disease features at baseline, according to expert opinion (disease duration, VAS of lung impairment at baseline, mRSS, and disease activity score). *p*-value <0.05 was considered statistically significant. The data analysis was performed using IBM SPSS version 26.0 (IBM Corp.) and FlowJo software 8.7.1 (Treestar, Ashland, OR).

## Results

### General characteristics of patients at baseline

Detailed characteristics of the study population at inclusion are summarised in Table 1: 54.2% of SSc patients had ILD at baseline with a median disease duration of 3 years. There were no differences between the two subgroups of SSc patients (with and without ILD at inclusion) regarding general demographic data, lifestyle, comorbidities, age at disease onset, and disease duration. However, ILD was most frequently associated with dSSc, ATA positivity, higher mRSS, decreased predictive value of FVC% and DLCO%, and the presence of sclerodactyly, pitting scars, active disease, late NVC pattern, higher functional impairment, and more severe respiratory symptoms at baseline.

**Table 1:** Characteristics of study population at baseline.

Variables	SSc_ILD+ (*n* = 32)	SSc_ILD− (*n* = 27)	Controls (*n* = 46)
Demographic characteristics			
Age, years (mean ± SD)	53.8 ± 10.9	55.4 ± 11.6	51.3 ± 9.3^a^
Sex, female/male, *n*	26/6	26/1	41/5^b^
BMI, kg/m^2^ (mean ± SD)	23.3 ± 3.0	23.9 ± 4.0	23.1 ± 2.8^a^
Current smokers, *n* (%)	10 (31.3)	6 (22.2)	9 (19.6)^b^
Arterial hypertension, *n* (%)	19 (61.3)	12 (38.7)	/0/
Disease characteristics			
Cutaneous subtype, *n* (%)			
Limited	13 (40.6)	25 (92.6)	
Diffuse	19 (59.4)^*^	2 (7.4)^b^	
Autoantibody status, *n* (%)			
Anti-centromere Ab	8 (25.0)	15 (55.6)^b^	
Anti-topoisomerase I Ab	19 (59.4)^*^	7 (25.9)^b^	
Age at disease onset	47.5 ± 12.9	48.6 ± 10.7^c^	
Disease duration, years, [median (min–max)]	3 (0–29)	6 (0–24)^d^	
mRSS, (median /IQR)	15 (3–31)^*^	6.5 (3–17.5)^d^	
FVC % predicted, (mean ± SD)	88.2 ± 15.7^*^	105.3 ± 18.6^c^	
DLCO% predicted, (mean ± SD)	55.7 ± 13.0^*^	77.0 ± 11.4^c^	
sPAP mmHg, (mean ± SD)	31.8 ± 8.2	29.1 ± 6.2^c^	
Telangiectasia present, *n* (%)	20 (64.5)	18 (69.2)^b^	
Sclerodactyly, *n* (%)	26 (81.3)^*^	11 (40.7)^b^	
Pitting scars, *n* (%)	22 (71)^*^	11 (42.3)^b^	
Contracture, *n* (%)	12 (38.7)	4 (15.4)^b^	
Digital ulcers ever, *n* (%)	23 (74.2)	16 (61.5)^b^	
Active disease, *n* (%)	23 (74.2)^*^	5 (19.2)^b^	
NVC *n* (%)			
Early	6 (19.4)	8 (30.8)	
Active	11 (35.5)	14 (53.8)	
Late	14 (45.2)^*^	4 (15.4)^b^	
SHAQ, [median (min–max)]	0.5 (0–2.6)^*^	0.25 (0–0.9)^d^	/
VAS lung, mm, [median (min–max)]	11 (0–90)^*^	0 (0–20)^d^	
Treatment, *n* (%)			
I. Ongoing treatment			
Glucocorticoids	12 (37.5)	10 (37.0)^b^	/
Metothrexate	5 (15.6)	9 (33.3)^b^	
Azathioprine	6 (19.4)	3 (11.5)^b^	
Mycophenolate mofetil	2 (6.3)	0 (0)^f^	
Antimalarial agents	1 (3.1)	4 (14.8)^f^	
II. Previous treatment			
Cyclophosphamide	17 (53.1)	7 (25.9)^b^	
Laboratory parameters			
Cholesterol, mmol/L, (mean ± SD)	6.5 ± 1.6	7.1 ± 1.6	6.1 ± 2.1^a^
Triglycerides, mmol/L, (mean ± SD)	1.6 ± 0.6	1.4 ± 0.7	1.3 ± 0.6^e^
CRP, mg/L, [median (min–max)]	6.7 (0.3–30.6)^*,#^	3.9 (0–16.4)	2.5 (0–7.1)^e^
IL-6, pg/mL, [median (min–max)]	6 (0–46)^*,#^	1.6 (0–11)^#^	0 (0–3)^e^
Platelets,10^9^ /L, (mean ± SD)	240.9 ± 67.8	232.7 ± 53.3	256.7 ± 50.3^a^
Leucocytes, 10^9^ /L, (mean ± SD)	6.8 ± 1.9	6.0 ± 1.7	6.2 ± 0.9^a^

Differences across three groups were calculated with either ^a^one-way ANOVA, ^b^*χ*^2^ test, or ^e^Kruskal–Wallis with *post hoc* analysis. *p*-values were adjusted by the Bonferroni correction for multiple testing.

Differences between the two ILD groups were analysed by ^b^*χ*^2^ test, ^c^Student’s *t*-test, ^d^Mann Whitney *U* test, or ^f^Fisher test.

^*^p < 0.05 observed between ILD and non ILD group.

^#p < 0.05 observed between either the ILD or non-ILD group and the control group.^

ILD, interstitial lung disease; Ab, autoantibody; BMI, body mass index; mRSS, modified Rodnan Skin Score; FVC, forced vital capacity; DLCO, diffusing capacity for carbon monoxide; sPAP, systolic pulmonary artery pressure; RP, Raynaud’s phenomenon; NVC, nailfold videocapillaroscopy; SHAQ, Scleroderma Health Assessment Questionnaire; VAS, Visual Analogue Scale; CRP, C reactive protein; IL6, interleukin 6.

Concerning ongoing immunosuppressive therapy at inclusion, cases with and without ILD did not differ in the frequency of use. The cumulative dose of CyC [12 g (5.5–20) vs. 12.8 g (4.8–15)], and the daily doses of GCs [5 mg (2.5–10) vs. 5 mg (2.5–5)] and the GK treatment duration (median 18 vs. 24 months, *p* > 0.05), were similar in the two subgroups.

### The levels of EVs and correlation with the presence of ILD, lung function tests, respiratory symptoms, immunosuppressive therapy, and smoking status at inclusion

**Figure 1:**
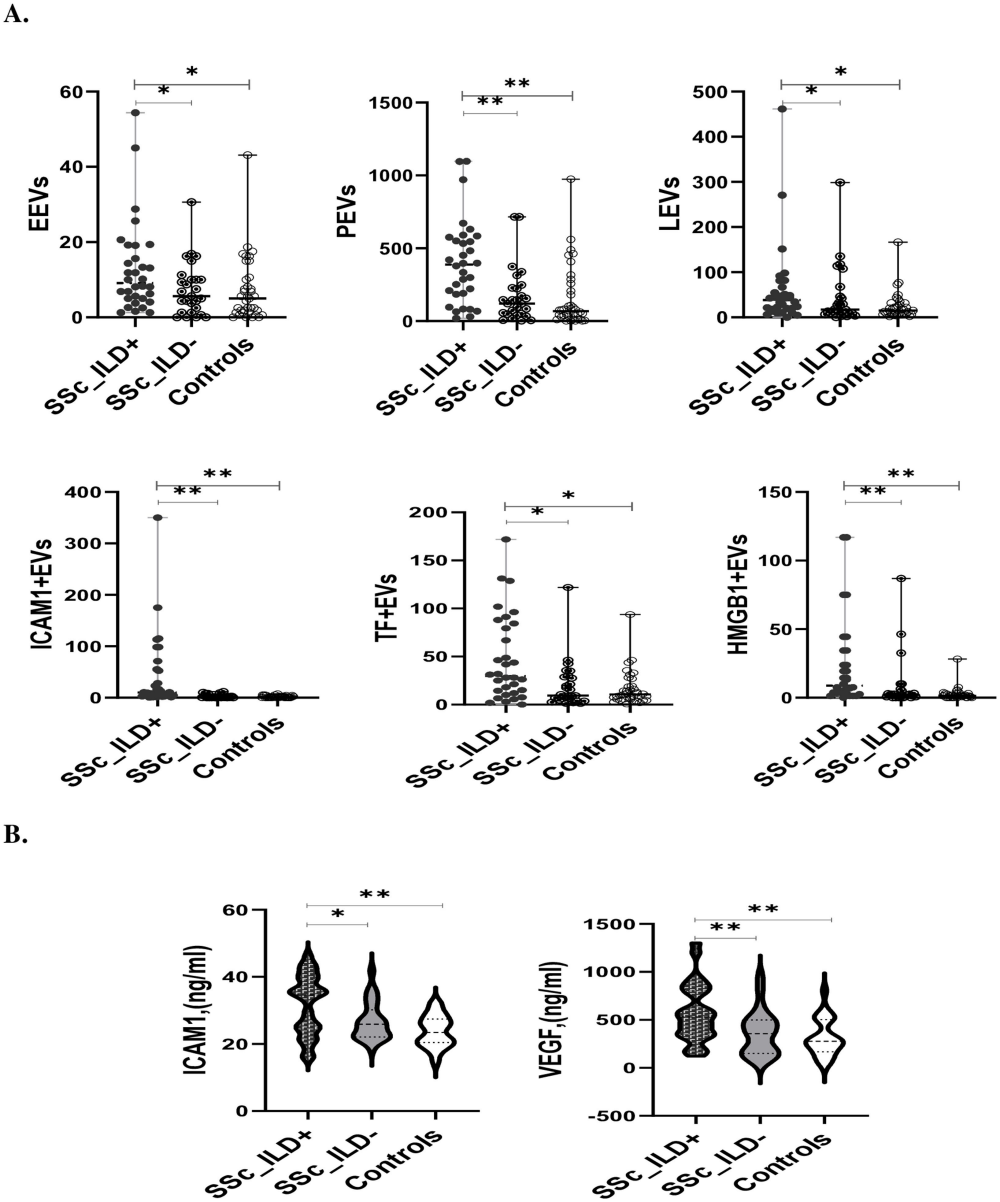
The levels of EVs **(A)** and endothelial injury markers **(B)** in SSc patients with and without ILD and controls. EEVs, endothelial extracellular vesicles; PEVs, platelet extracellular vesicles; LEVs, leucocytes extracellular vesicles; ICAMI + EVs, extracellular vesicles expressing Intercellular adhesion molecule 1; TF + EVs, extracellular vesicles expressing tissue factor; HMGB1 + EVs extracellular vesicles expressing high mobility group box 1; ILD, interstitial lung disease. ^*^p < 0.05 and ^**^p < 0.001.

At baseline, the concentrations of plasma levels of all investigated EVs (Figure 1A), as well as serum levels of endothelial injury (Figure 1B) and inflammatory markers (Table 1), were increased in SSc-ILD cases compared to those without ILD and controls. Moreover, therapy naïve SSc-ILD cases (*n* = 12/32) differed remarkably only in levels of ICAM1 + EVs compared to treated patients [50.8 (2.5–359.0) vs. 6.6 (0.6–71.3), *p* = 0.01].

In the SSc cohort, the levels of the majority of circulating EVs was significantly inversely correlated with FVC% and DLCO% (Table 2A). In SSc-ILD, only ICAM1 + EVs and TF + EVs were significantly negatively correlated with the FVC%, while none of the investigated EVs exhibited a significant correlation with DLCO% (Table 2B).

**Table 2:** Correlation between circulating EVs with pulmonary function tests and patient reported outcomes within the cohort of patients with (A) systemic sclerosis or (B) SSc-ILD at baseline.

	(A) SSc			(B) SSc-ILD		
	FVC (%)	DLCO (%)	VAS lung (mm)	FVC (%)	DLCO (%)	VAS lung (mm)
EEVs/μL	−0.3^*^	−0.2	0.2	−0.2	−0.2	0.2
LEVs/μL	−0.4^**^	−0.1	0.3^*^	−0.3	−0.2	0.4^*^
PEVs/μL	−0.1	−0.4^**^	0.5^***^	−0.1	−0.1	0.4^*^
ICAM1 + EVs/μL	−0.4^**^	−0.5^***^	0.5^***^	−0.4^*^	−0.2	0.4^*^
TF + EVs/μL	−0.4^**^	−0.3^*^	0.4^**^	−0.4^*^	−0.2	0.4
HMGB1 + EVs/μL	−0.3^*^	−0.3^*^	0.4^**^	−0.3	−0.1	0.2

FVC, forced vital capacity; DLCO, diffusing capacity for carbon monoxide; VAS, Visual Analogue Scale; EEVs, endothelial extracellular vesicles; PEVs, platelet extracellular vesicles; LEVs, leucocytes extracellular vesicles; ICAM1 + EVs, extracellular vesicles expressing intercellular adhesion molecule 1; TF + EVs, extracellular vesicles expressing tissue factor; HMGB1 + EVs extracellular vesicles expressing high mobility group box 1, SSc, systemic sclerosis; ILD, interstitial lung disease. *p < 0.05, **p < 0.01, ***p < 0.001.

In terms of the potential effect of smoking on EVs levels, a subgroup analysis showed that even though SSc-ILD patients with smoking habits had elevated levels of PEVs, LEVs, and ICAM1 + EVs compared to non-smokers, a statistically significant difference was not observed (data not shown).

Apart from EEVs, all other EVs were positively significantly correlated with the alteration of lung symptoms within SSc cases (Table 2A). Of 25 SSc patients with respiratory symptoms at baseline, 80% belong to the SSc-ILD group. Increased concentrations of PEVs [436 (83.13–1,096) vs. 142 (18.7–1,096), *p* = 0.007], LEVs [41.1 (0–461.3) vs. 17.4 (0.8–298.4), *p* = 0.03] and ICAM1 + EVs [16.1 (2.13–175.0) vs. 3.5 (0.6–359.0), *p* = 0.012] were found in SSc-ILD patients with respiratory symptoms compared to asymptomatic. Moreover, PEVs, LEVs, and ICAM1 + EVs showed a significant positive correlation with a worsening of lung symptoms (Table 2B). Among SSc-ILD symptomatic patients, 35% were smokers. Smokers did not differ in any of the investigated EVs in comparison to non-smokers (data not shown). Further on, 7/20 SSc-ILD patients with respiratory symptoms were therapy naïve and they had significantly increased levels of only ICAM1 + EVs compared to those on immunosuppressive therapy [81.3 (6.5–175.0) vs. 10.6 (2.1–71.3, *p* = 0.006)].

### The levels of investigated EVs in the SSc-ILD cohort in relation to proinflammatory and endothelial injury markers and disease activity

A strong, significant positive correlation was observed only between ICAM1 + EVs and serum levels of proinflammatory markers and VEGF. At the same time, no relation was detected between all investigated EVs and serum levels of ICAM1 (Supplementary Table 1) among cases with SSc-ILD. Conversely, no correlation was found between EVs and serum levels of all investigated inflammatory and endothelial injury markers in cases without ILD (data not shown).

Within the SSc-ILD cohort, 74.2% of patients had active disease. Of note, SSc-ILD patients with active disease had significantly altered concentrations of ICAM1 + EVs and PEVs compared to patients without active disease (*p* < 0.05). Plasma levels of EEVs, LEVs, and ICAM1 + EVs were positively moderately correlated with disease activity score (*ρ* = 0.4; *ρ* = 0.4; *ρ* = 0.5; *p* < 0.05, respectively). Moreover, in SSc-ILD cases with active disease and cases naïve to immunosuppressive therapy (8/23), only ICAM1 + EVs levels were found to be remarkably elevated [9.9 (0.6–359.0) vs. 1.25 (0–11.9), *p* < 0.001].

### Levels of investigated EVs in the SSc-ILD cohort in relation to other disease features

Notably, elevated levels of EEVs and ICAM1 + EVs (*p* < 0.05 for both variables) were detected, in patients with dSSc, when compared to lSSc [11.9 (3.8–54.4) vs. 6.3 (1.3–28.8); 20.5 (1.3–359.0) vs. 5.0 (0.6–175.0)], those with ATA positive autoantibody status [10.2 (2.5–54.4) vs. 6.9 (1.3–28.8); 10.6 (1.3–359.0) vs. 6.5 (0.6–175.0)], cases with early stage of disease compared to late stage [13.3 (1.3–54.4) vs. 5.6 (1.6–14.4); 23.1 (0.6–359.0) vs. 5 (1.3–20.5)], and cases with sclerodactyly [11.1 (1.3–54.4) vs. 2.6 (1.3–13.3); 16.1 (0.6–359.0) vs. 3.4 (1.3–10.6)]. A moderate negative correlation was also found between both EEVs and ICAM1 + EVs and disease duration (*ρ* = 0.5, *p* < 0.05, respectively). No significant association was found between EV levels and other recorded clinical features. Concerning therapeutic modalities, only subjects previously treated with Cyc showed lower concentrations of ICAM1 + EVs [5 (0.63–71.3) vs. 23.1 (2.5–359.0), *p* = 0.005]. However, no significant correlation was observed between a cumulative dose of Cyc or a daily dose of glucocorticoids with any of the investigated EVs (data not shown).

### Independent blood risk factors with the presence of SSc-ILD

Univariate logistic regression analysis with continuous laboratory variables that previously showed a significant association with SSc-ILD are presented in Supplementary Table 2A. Multivariate logistic regression analysis adjusted for VEGF and IL6 and revealed an independent association between PEVs (OR 1.004, 95% CI 1.001–1.01) and ICAM1 + EVs (OR 1.3, 95% CI 1.1–1.5) and the presence of ILD (Supplementary Table 2B). Even when CRP was added to the previous enter model, ICAM1 + EVs and PEVs remained independently associated with ILD (OR 1.3, 95% CI 1.1–1.6, OR 1.004, 95% CI 1.001–1.01) (Supplementary Table 2B). Of note, neither VEGF, IL-6, or CRP remained significantly associated with SSc-ILD in any of the models analysed above.

In addition, multivariate linear regression showed that VEGF was the only serum marker independently associated with ICAM1 + EVs within the SSc-ILD group (*β* = 0.7, 95% CI 0.7–2.9, *p* < 0.01), thus explaining 40% of the variability.

**Figure 2:**
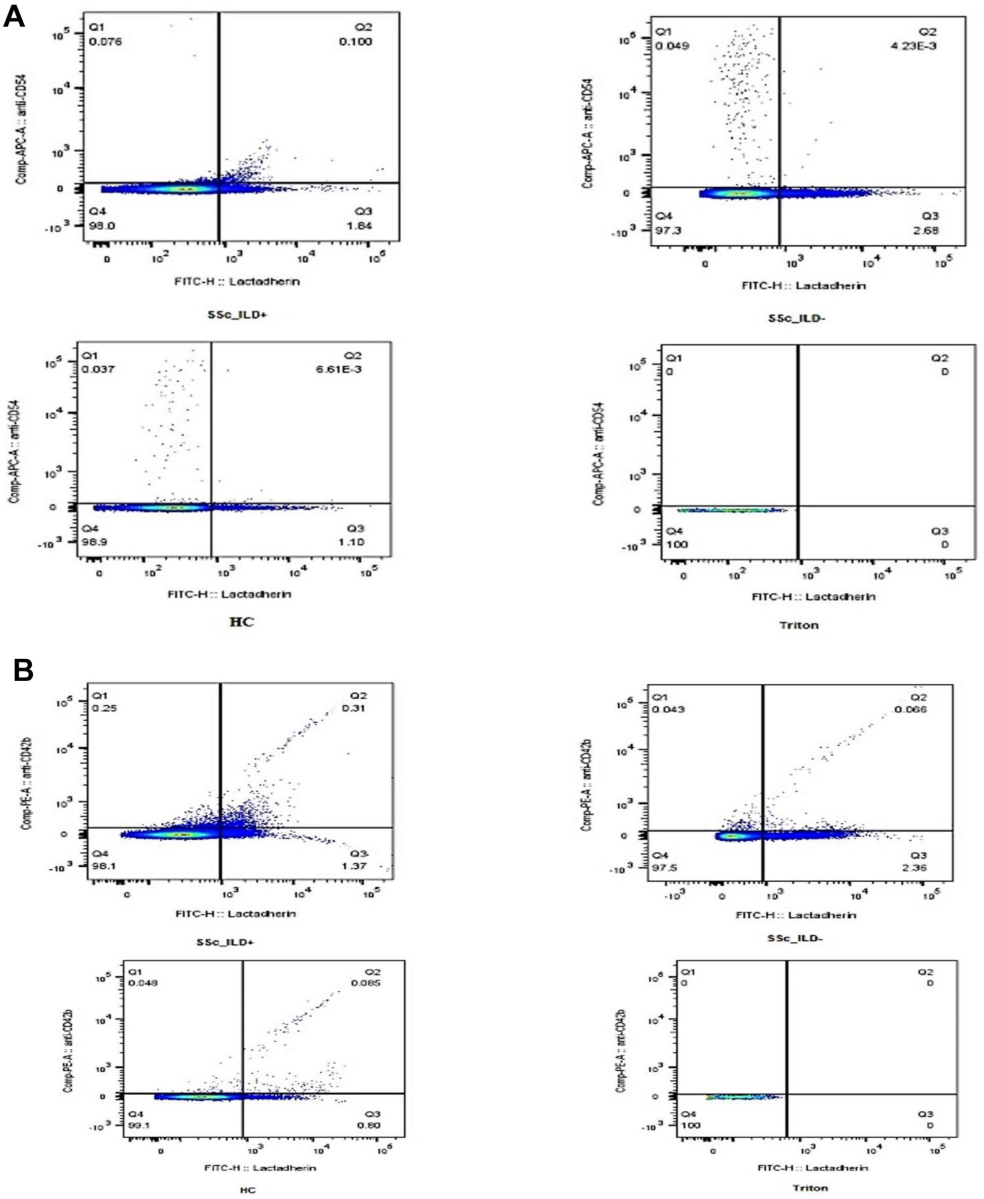
Representative samples of PS + EVs expressing ICAM1 and PEVS. EVs positive for lactadherin (PS + EVs) and ICAM1 in samples with SSC-ILD, without SSC ILD, controls and Triton **(A)**. EVs positive for lactadherin (PS + EVs) and originated from platelets (PEVs) in samples with SSC-ILD, without SSC ILD, controls, and with Triton **(B)**. EVs, extracellular vesicles; ILD, interstitial lung disease; SSc, systemic sclerosis.

Representative dot plots for ICAM1 + EVs and PEVs are presented in Figure 2 within the Q2 window.

### Clinical characteristics of SSc patients during 3 years of follow up

During the 3 years of follow-up, none of the SSc patients without ILD at baseline had new-onset ILD. In those with ILD, progression was observed in 12/32 (37.5%) cases, of which 58% had an ATA + profile and dSSc disease type with less than 1 year of disease duration: the estimated median time to progression was 18 (6–36) months. The majority of PF-ILD cases were observed in the first year of follow-up (*n* = 6/12), while the frequency of progression was the same within the second (25%) and third (25%) years. Progression of ILD, defined as a decline of FVC of ≥10%, was observed in (7/12) 60% of cases. ILD progression was highly associated with the presence of dSSc, active disease, and worser lung symptoms at inclusion. There were no differences between other demographic and clinical features between patients with and without PF-ILD, as presented in Table 3. All therapy naïve ILD patients (*n* = 12/32) at inclusion, of which 83% were newly diagnosed cases with less than 1 year of SSc duration, started treatment with CyC soon after enrolment, and the majority (*n* = 7/12) developed PF-ILD over the FU period.

**Table 3:** Characteristics of patients at baseline who experienced progressive phenotype of ILD over the follow-up period.

Variables	PF-ILD (*n* = 12)	Non-PF-ILD (*n* = 20)
Demographic characteristics		
Age, years, (mean ± SD)	53.1 ± 10.9	54.3 ± 11.1^a^
Sex, female/male, *n*	10/2	16/4^b^
BMI, kg/m^2^ (mean ± SD)	22.3 ± 1.9	23.9 ± 3.4^a^
Current smokers, *n* (%)	13 (33.3)	3 (15.8)^b^
Arterial hypertension, *n* (%)	5 (41.7)	14 (70.0)^b^
Disease characteristics		
Cutaneous subtype, *n* (%)		
Limited	2 (16.7)	11 (55.0)^b^
Diffuse	10 (83.3)^*^	9 (45.0)^b^
Autoantibody status, *n* (%)		
Anti-centromere Ab	2 (16.7)	6 (30.0)^c^
Anti-topoisomerase I Ab	7 (58.3)	12 (60.0)^b^
Age at disease onset	47.9 ± 12.6	46.9 ± 13.6^a^
Disease duration, years, [median (min–max)]	0 (0–29)^#^	3.5 (0–19)^a^
mRSS, [median (min–max)]	16 (4–31)	15 (3–31)^a^
FVC % predicted, (mean ± SD)	87.6 ± 18.3	88.6. ± 14.5^a^
DLCO% predicted, (mean ± SD)	52.7 ± 10.9	57.5. ± 14.1^a^
sPAP mmHg, (mean ± SD)	34.4 ± 11.5	30.4 ± 5.6^a^
Telangiectasia present, *n* (%)	7 (63.6)	13 (65.0)^b^
Sclerodactyly, *n* (%)	12 (100)	14 (70)^b^
Contracture, *n* (%)	6 (54.4)	6 (30)^b^
Pitting scars, *n* (%)	8 (72.7)	14 (70)^b^
Active disease, *n* (%)	12 (100)^*^	12 (52)^b^
NVC, *n* (%)		
Early	2 (18.2)	4 (20.0)
Active	2 (18.2)	9 (45.0)
Late	7 (63.6)	7 (35.0)^c^
SHAQ, [median (min–max)]	0.9 (0–2.6)^*^	0.4 (0–1.5)^a^
VAS lung, mm, [median (min–max)]	34 (0–90)^*^	4 (0–80)^a^
Treatment, *n* (%)		
I. Ongoing treatment		
Glucocorticoids	5 (41.7)	7 (35.0)^c^
Metothrexate	1 (8.3)	4 (20)^c^
Azathioprine	1 (8.3)	5 (26.3)^c^
II. Previous treatment		
Cyclophosphamide	4 (33.3)	13 (65.0)^b^
Laboratory parameters		
Cholesterol, mmol/L, (mean ± SD)	6.2 ± 1.9	6.6 ± 1.5^a^
Triglycerides, mmol/L, (mean ± SD)	1.6 ± 0.7	1.6 ± 0.6^a^
CRP, mg/L, [median (min–max)]	10.5 (5.4–30.6)^**^	3.7 (0.3–13.2)^a^
IL-6, pg/mL, [median (min–max)]	8.2 (2–46)^**^	0 (0–22)^a^
Platelets,10^9^/L, (mean ± SD)	266.1 ± 76.9	228.4 ± 61.0^a^
Leucocytes, 10^9^ /L, (mean ± SD)	7.2 ± 1.9	6.5 ± 1.9^a^
ICAM 1, ng/mL, (mean ± SD)	32.8 ± 7.35	31.9 ± 9.51^a^
VEGF, ng/mL, [median (min–max)]	781.5 (444–1,300)^**^	371 (123–1,200)^a^

Group differences were calculated using a Mann Whitney, ^b^*χ*^2^ test or ^c^Fisher exact test.

^*^p < 0.05, ^**^p < 0.001, and ^#^*p* = 0.1.

ILD, interstitial lung disease; Ab, autoantibody; BMI, body mass index; mRSS, modified Rodnan Skin Score; FVC, forced vital capacity; DLCO, diffusing capacity for carbon monoxide; sPAP, systolic pulmonary artery pressure; RP, Raynaud’s phenomenon; NVC, nailfold videocapillaroscopy; SHAQ, Scleroderma Health Assessment Questionnaire; VAS, Visual Analogue Scale; CRP, C reactive protein; IL6, interleukin 6, ICAM1, intercellular adhesion molecule 1; VEGF, vascular endothelial growth factor.

### The levels of investigated EVs in relation to PF-ILD onset

**Figure 3:**
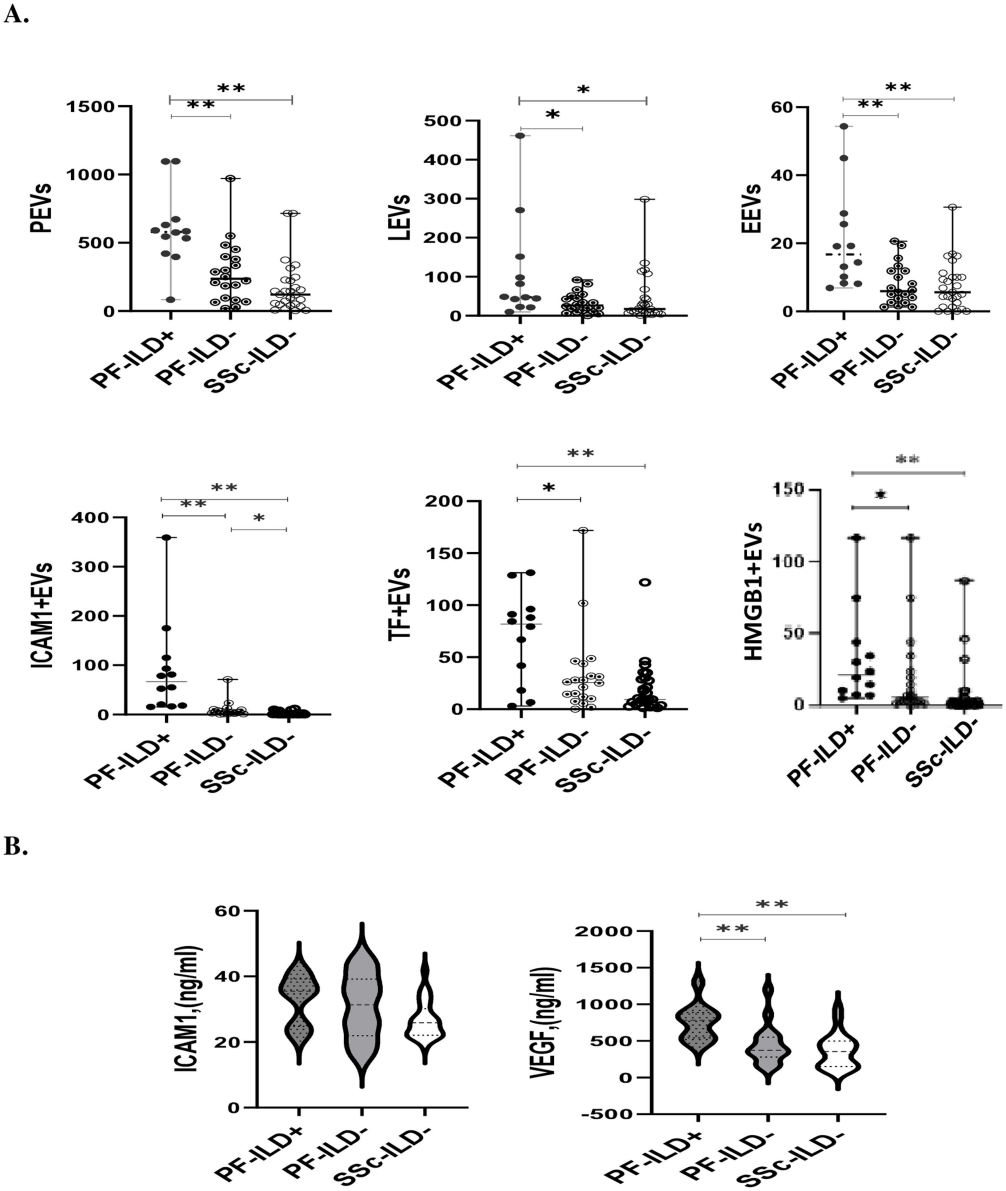
The levels of EVs **(A)** and endothelial injury markers **(B)** in SSc patients with and without PF-ILD onset over 3 years of follow-up. EEVs, endothelial extracellular vesicles; PEVs, platelet extracellular vesicles; LEVs, leucocytes extracellular vesicles; ICAM1 + EVs, extracellular vesicles expressing intercellular adhesion molecule 1; TF + EVs, extracellular vesicles expressing tissue factor; HMGB1 + EVs, extracellular vesicles expressing high mobility group box 1; PF-ILD, progressive interstitial lung disease. ^*^p < 0.05 and ^**^p < 0.001.

Figure 3 and Table 3 present the plasma levels of all investigated EVs and serum markers in relation to PF-ILD onset. The highest concentrations of all circulating EVs, IL6, and VEGF levels were found in patients with PF-ILD when compared to those without progression and those with SSc cases that remained without ILD over FU.

**Figure 4:**
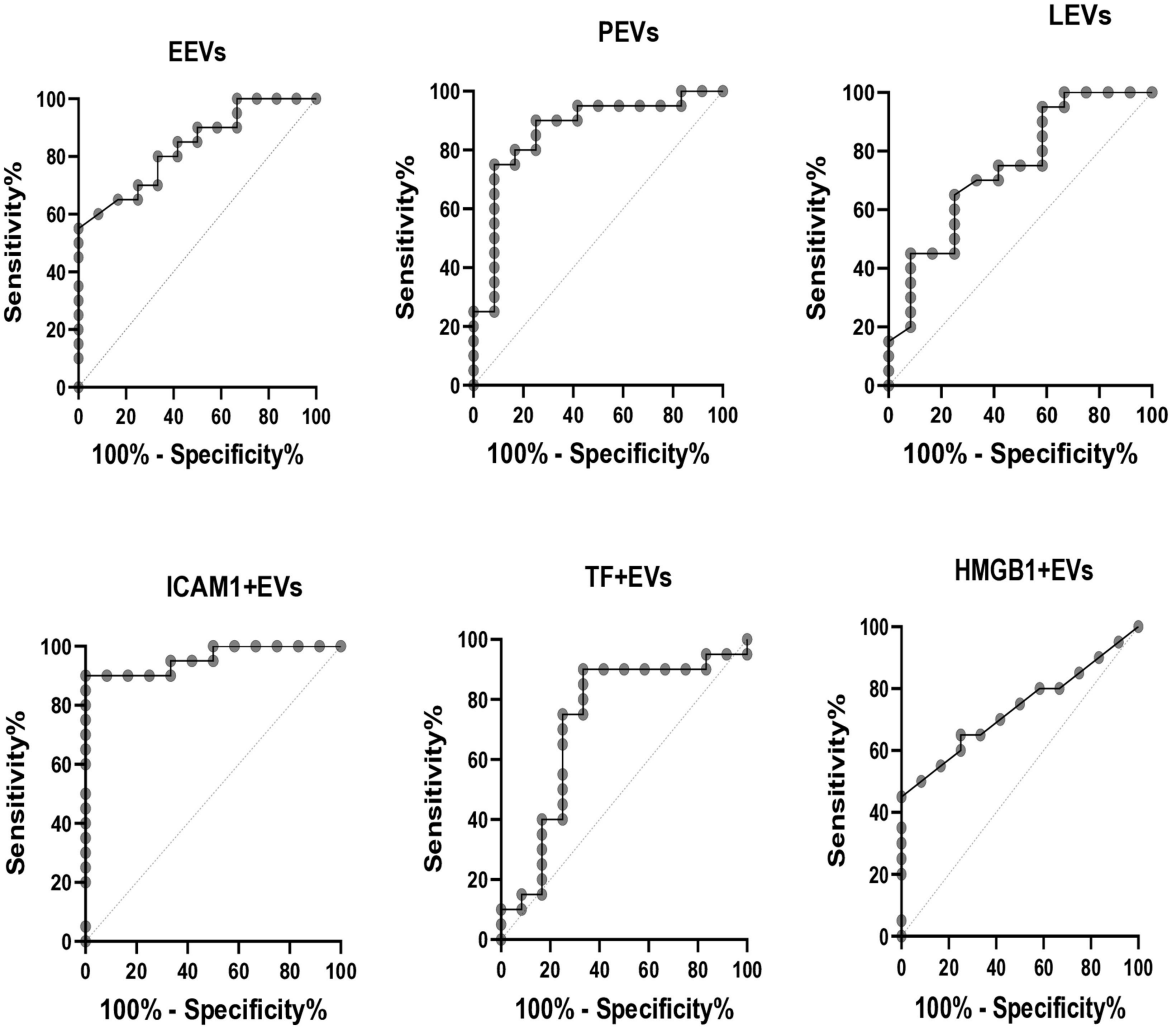
ROC curves for PF-ILD = 1. EEVs, endothelial extracellular vesicles; PEVs, platelet extracellular vesicles; LEVs, leucocytes extracellular vesicles; ICAM1 + EVs, extracellular vesicles expressing intercellular adhesion molecule 1; TF + EVs, extracellular vesicles expressing tissue factor; HMGB1 + EVs, extracellular vesicles expressing high mobility group box 1.

ROC analysis was used to assess the validity of EVs in identifying patients with PF-ILD (Figure 4). Areas under the curve (AUCs) (95% CI) were the highest for ICAM1 + EVs and PEVs [0.9 (0.9–1), *p* < 0.01, respectively], followed by EEVs [0.8 (0.7–0.9), *p* < 0.01], LEVs [0.7 (0.6–0.9), *p* < 0.05], TF + EVs [0.7 (0.5–0.9), *p* < 0.05], and HMGB1 + EVs [0.7 (0.6–0.9), *p* < 0.05].

### Correlation between levels of EVs and other laboratory and clinical features within the PF-ILD cohort

Within cases with ILD progression, a significant correlation was found between ICAM1 + EVs and VEGF (*ρ* = 0.8, *p* < 0.01), IL6 (*ρ* = 0.8, *p* < 0.01), and VAS respiratory symptoms alteration (*ρ* = 0.6, *p* < 0.05). Furthermore, apart from the inverse strong relation between ICAM1 + EVs and disease duration (*ρ* = 0.8, *p* < 0.01), no other significant correlation was found between investigated EVs and continuous clinical variables, including serum ICAM1 levels, CyC dosage, and GCs at inclusion (data not shown).

### Predictive value of EVs in PF-ILD onset

A multivariate Cox regression analysis adjusted for VEGF and IL6 revealed that only EVs expressing ICAM1 (HR 1.1, 95% CI 1.01–1.1) had independent predictive value for PF-ILD onset over 3 years of FU. Adding CRP to the same model did not change the significance of the results. The final forward model, including disease duration, VAS lung impairment, mRSS, and disease activity score, along with previously tested laboratory data, showed that ICAM1 + EVs (HR 1.1, 95% CI 1.02–1.1), and IL6 (HR 1.1, 95% CI 1.03–1.2) were independent predictors of SSc-ILD progression.

### Patient characteristics with the highest ICAM1 + EVs

**Figure 5:**
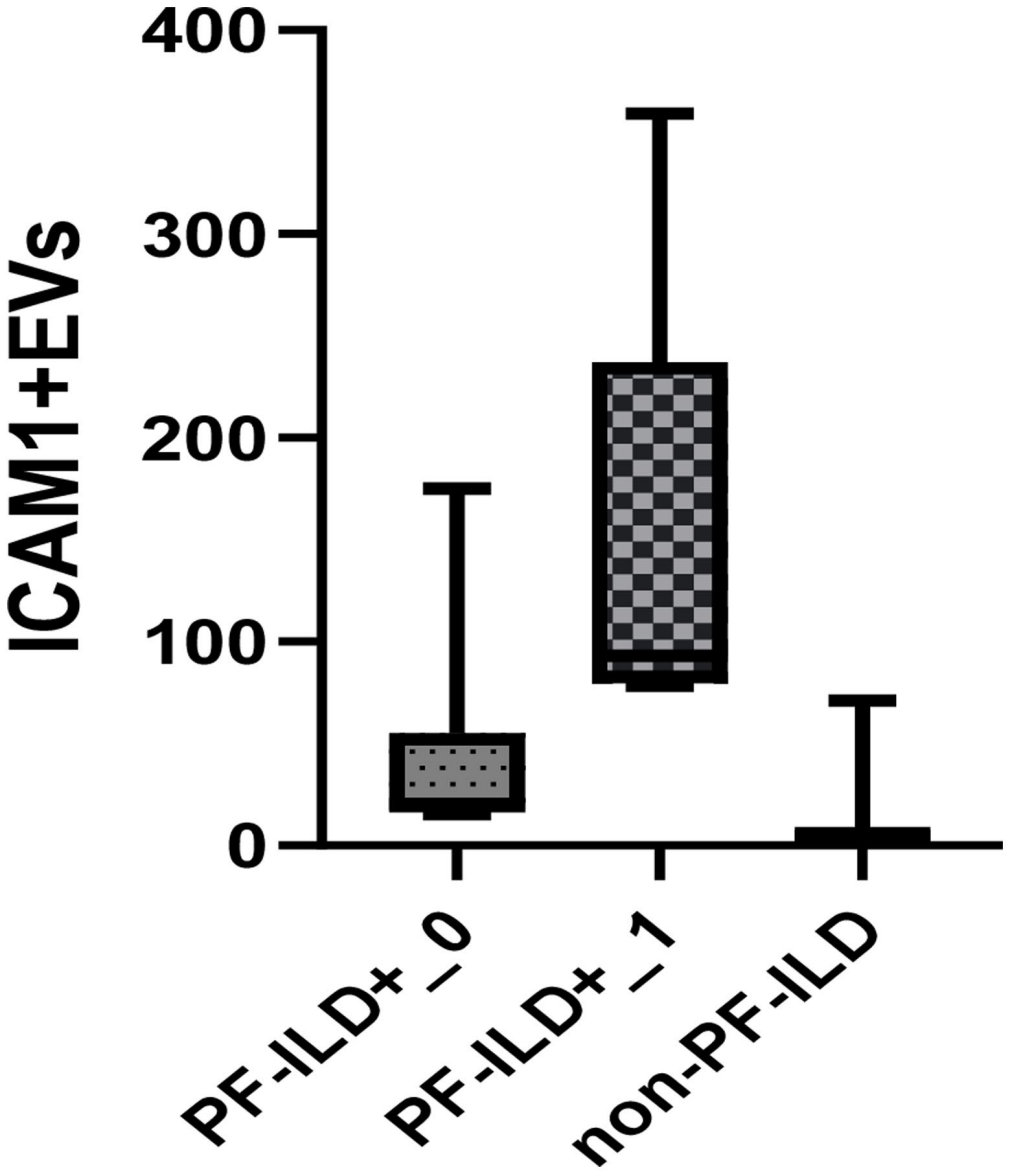
Baseline EVs ICAM1 concentration in PF-ILD+_1 group (disease duration less than 1 year, dSSc, active disease, FVC <80%, DLCO% <70%, symptomatic patients), PF-ILD_0 (other PF-ILD patients than in group PF-ILD+_1) and non-PF-ILD over a 3-year follow-up period.

Of note, the patient with the highest level of ICAM1 + EVs at baseline was a 36-year old male, smoker, with an ATA + profile. He had the dSSc disease subtype, both FVC% and DLCO% <70% of predicted, and disease duration of 11 months at inclusion. He developed PF-ILD after 6 months, with FVC decline of >10%, although CyC therapy was initiated soon after enrolment. In line with this, a subgroup of patients amounting to 42% of the PF-ILD cohort, which were characterised by dSSc, active disease, disease duration less than 1 year, decreased LFTs (FVC <80% and DLCO% <70%), respiratory symptoms, and immunosuppressive therapy naïve, had a notably higher concentration of ICAM1 + EVs and experienced PF-ILD already at 6 (6–12) months of follow up (Figure 5).

## Discussion

Our study is the first to show increased levels of different circulating EVs in the plasma of SSc patients characterised by a functional progression of SSc-ILD. Moreover, EVs expressing ICAM1 correlated inversely with FVC within the SSc-ILD cohort, mirroring the severity of ILD on HRCT, and were independently associated with the onset of ILD progression in these patients. Therefore, we postulated that EVs may have a pivotal role in PF-ILD pathogenesis by mediating the complex interplay between endothelial injury, inflammation, activation of coagulation, and oxidative stress.

It is well known that ILD may affect more than 65 of SSc patients, with mortality from 6 to 33% (1, 3). However, ILD progression is expected in almost 40% of untreated patients over 3 years, or 67% over 5 years, with a worse prognosis and a mortality rate of around 15% in the initial one-year period (20–22). The cumulative incidence of PF-ILD during the 3 years of follow-up in our ILD cohort was around 40%. These patients were naïve to recently approved SSc-ILD targeted therapies (26–28), and almost 40% of cases were never treated with any of the immunosuppressive modalities recommended by EULAR (29) prior to inclusion. Further along in the study, our SSc cases had no comorbidity besides arterial hypertension. Therefore, we assumed that our results could, to some extent, reflect the natural course of SSc-related ILD in a real-life clinical setting.

Although various markers have been explored, there are still no reliable and accessible biomarkers to predict the course of SSc-ILD (5). Novel experimental SSc data demonstrate direct fibrotic features of EVs on fibroblasts by inducing type I collagen expression (10). However, to date, few clinical studies have explored the association of EVs originating from different cells with lung affection in SSc (10, 12–14). The largest study to date (*n* = 33) showed increased concentrations of total EVs and EEVs, but no PEVs, in HRCT-confirmed ILD in SSc patients (10). On the other hand, elevated PEVs and monocyte-derived EVs were found in SSc patients with interstitial pneumonia without radiographic confirmation of lung fibrosis (13). Lastly, enhanced levels of activated EEVs were observed in SSc patients with lung fibrosis (12). This is in contrast to the most recent study by de Oliveira et al. (14), which did not show an association between EEVs, PEVs, or monocyte-derived EVs and SSc-ILD.

Compared to the previously mentioned studies, EV phenotyping was different in our study. We demonstrated elevated levels of all investigated circulating EVs (EEVs, PEVs, LEVs, ICAM1 + EVs, TF + EVs, and HMGB1 + EVs) in patients with SSc-ILD compared to patients without SSc-ILD or controls, as well as an inverse correlation with the respiratory function tests at baseline. Further elevation of all EV levels was associated with PF-ILD over the follow-up period, suggesting that EV levels are involved in the pathogenesis, severity, and progression of lung fibrosis. It has been previously described that EEV may contribute to the migration of lung fibroblasts in idiopathic pulmonary fibrosis (30). EEVs and PEVs were associated with endothelial injury markers (E- and P-selectin) in SSc, implicating their role in the pathogenesis of vasculopathy (12). PEVs released from activated platelets can induce leucocyte activation, generation of LEVs, and TF expression, causing the activation of fibroblasts in SSc (13). EVs exposing TF may further activate coagulation cascade and platelet aggregation (31), along with the ability to transfer TF among EVs (32). PEVs expressing HMGB1 were found to be linked with vascular injury in SSc, while experimental data showed that they also had a role in neutrophil activation, consequently inducing endothelial injury and fibrosis in mice (8, 33). In summary, our results may further contribute to the understanding of the vital role EVs play in endothelial and alveolar injury, inflammation, initiation of hypercoagulable state, and oxidative stress on the way to progressive lung fibrosis development.

The most prominent result of our study is the independent association of ICAM1 + EVs with SSc-ILD onset and progression. However, this finding is not surprising, since ICAM1 has been extensively studied in lung injury models, exhibiting dual pro-inflammatory and fibrotic features by driving leukocyte recruitment and transmigration, endothelial to mesenchymal transition, and regulating the accumulation of profibrotic cells to the lungs (34–36). The severity of pulmonary fibrosis has been shown to be directly impacted by the number of endothelial cells expressing ICAM1 (37). Recently, soluble (s) ICAM1 in serum was proposed as a biomarker of ILD in SSc (38). We found significantly elevated sICAM1 in patients with SSc-ILD, but not in cases with a progressive pattern. Although sICAM1 may reflect ICAM1 expression on endothelial cells (39), the level of EVs expressing ICAM1 was not related to sICAM1 in our cohort, irrespective of lung-affected subgroups, suggesting that assessing adhesion molecules on EVs could be a more sensitive and reliable method.

Furthermore, we found a strong positive correlation between ICAM1 + EVs and well-known mediators of SSc lung fibrosis (IL6 and VEGF) (7) within both ILD and PF-ILD subgroups. Additionally, VEGF explained 40% of ICAM1 + EVs variability within our ILD cases. Indeed, *in vitro* data has shown that ICAM1 may directly provoke IL6 production (40), while VEGF can mediate ICAM1 upregulation via the PI3K/AKT/NO pathway (41). Interestingly, a positive correlation of ICAM1 + EVs with disease activity was observed within our SSC-ILD cases. However, a significant relation of ICAM1 + EVs was found only with the proinflammatory marker CRP among all other EUSTAR activity index components, reflecting the important role of this molecule in perpetuating inflammation. Moreover, even after adjusting for IL6, VEGF, and CRP, ICAM1 + EVs remained an independent predictive factor of ILD progression, indicating that ICAM1 + EVs could be a crucial proinflammatory/profibrotic signal pathway in lung fibrosis.

When interpreting our results, the clinical features of the cohort need to be considered. As expected, PF-ILD onset was more frequent in the dSSc subset, active disease, and altered respiratory symptoms (42). Notably, most PF-ILD cases had preserved FVC% at baseline, highlighting the need for the use of more sensitive screening and diagnostic tools (43) in daily practice. In our cohort, ICAM1 + EVs were found to be linked with well-recognised risk factors of ILD progression and mortality, including dSSc, shorter disease duration, active disease, and FVC% decline, further supporting their role in PF-ILD (44, 45). Concerning immunosuppressive therapy, we found that only cyclophosphamide could have a long-lasting impact on EVs in SSc ILD by decreasing the concentration of EVs expressing ICAM1. However, despite the initiation of cyclophosphamide treatment soon after inclusion in patients with newly diagnosed SSc-ILD who were naïve to any disease-modified treatment modalities, 60% developed PF-ILD, highlighting the importance of creating a personalised, targeted drug approach.

Our study has some limitations. First, the study has a small sample size; therefore, our results should be validated in a larger cohort of patients. A relatively low number of SSc ILD cases within the follow-up group did not allow us to include categorical variables in the multivariate Cox regression analysis, and we were not able to define a composite predictive model of PF-ILD development. We assessed only the functional progression of ILD. However, the exploration of HRCT progression would be very important. Furthermore, only Caucasians were enrolled, restricting the extrapolation of our results to other ethnicities. Tracking other clinical alterations over time that could impact SSc-ILD progression and their effect on EVs would add pivotal knowledge. Moreover, assessing EVs’ profile of patients who develop SSc-ILD during the study would provide a more comprehensive understanding of disease progression and mechanisms. Lastly, the measurement of EVs was not repeated at the time of PF-ILD onset. Therefore, a more extensive multinational longitudinal study should be performed following clinically well-characterised, ultimately therapy-naïve SSc-ILD patients from the time of diagnosis, assessing both functional and radiographic ILD progression and other clinical and laboratory alterations over time to confirm our preliminary findings. This was a pilot study assessing circulating EVs as predictive biomarkers of PF-ILD.

The bulk of the data present in the literature on ICAM1 may also raise the possibility that it may not only be a marker of the inflammatory activation of endothelial cells, but also being a leading factor in the pathogenesis. In fact, ICAM1 may amplify the inflammatory process (46), and implement the progression to fibrosis as shown in the model of bleomycin induced lung fibrosis (47). Thus, as a next step we plan to explore the *in vitro* profibrotic potential and mechanism of action of different EVs isolated from SSc-ILD patients to verify their role in progressive lung fibrosis pathogenesis.

In conclusion, the novelty of our study is the finding that circulating EVs may represent new predictive biomarkers alerting physicians of ILD progression in SSc. Our findings need to be replicated on larger longitudinal cohorts to verify the role of EVs as biomarkers in paving the road to new therapeutic avenues.

## Data Availability

The raw data supporting the conclusions of this article will be made available by the authors, without undue reservation.
