# Supplementary material for: Circulating extracellular vesicles as predictive biomarkers of progressive interstitial lung disease in systemic sclerosis—a prospective cohort study
Source: Front Med (Lausanne). 2025 Apr 30;12:1594201. doi: 10.3389/fmed.2025.1594201 (PMC12075389; doi:10.3389/fmed.2025.1594201)
Supplement: Supplementary file 1 [file Table_1.docx]

Supplementary Table 1.Correlation between circulating EVs with markers of inflammation and endothelial injury within the cohort of patients with SSc-ILD at the baseline.

|  | SSc -ILD | | |  |
| --- | --- | --- | --- | --- |
|  | CRP (mg/L) | IL6 (pg/ml) | VEGF (ng/ml) | ICAM1  (ng/ml) |
| EEVs/μL | 0.5^***^ | 0.5^**^ | 0.5^**^ | 0.2 |
| LEVs/μL | 0.3 | 0.1 | 0.4^*^ | 0.1 |
| PEVs/μL | 0.4^**^ | 0.5^**^ | 0.3 | 0.2 |
| ICAM1^+^EVs/μL | 0.6^***^ | 0.6^***^ | 0.6^***^ | 0.2 |
| TF^+^EVs/μL | 0.2 | 0.4^*^ | 0.4^*^ | 0.1 |
| HMGB1^+^EVs/μL | 0.3 | 0.2 | 0.6** | 0.1 |

The results are presented with a correlation coefficient. ^*^ p<0.05; ** p<0.01; ***p<0.001

Abbreviations: EEVs- endothelial extracellular vesicles; PEVs- platelet extracellular vesicles; LEVs- leucocytes extracellular vesicles; ICAM1^+^EVs- extracellular vesicles expressing Intercellular adhesion molecule 1; TF^+^EVs- extracellular vesicles expressing tissue factor; HMGB1^+^EVs extracellular vesicles expressing High mobility group box 1, SSc- systemic sclerosis; ILD- interstitial lung disease, CRP-C reactive protein; IL- interleukin; VEGF-Vascular Endothelial Growth Factor.

Supplementary Table 2. Univariate and multivariate logistic regression of laboratory risk factors for SSc-ILD

|  | **(A.)** Univariate logistic regression | | Multivariate logistic regression | | | |
| --- | --- | --- | --- | --- | --- | --- |
|  | | OR (95%CI) | **(B.)** aOR (95%CI) | | | **(C.)** aOR^1^ (95%CI) |
| EEVs/μL | 1.1 (1.01-1.2) | | 1.04 (0.9-1.1) | | | 1.04 (0.9-1.1) |
| LEVs/μL | 1.01(1.003-1.01) | | 1.0 (0.9-1.01) | | | 0.9 (0.9-1.01) |
| PEVs/μL | 1.01(1.002-1.01) | | 1.004(1.001-1.01 | | | 1.004(1.001-1.01) |
| ICAM1^+^EVs/μL | 1.3 (1.1-1.5) | | 1.3 (1.1-1.6) | | | 1.3 (1.1-1.6) |
| TF^+^EVs/μL | 1.03 (1.01-1.1) | | 1.02 (0.9-1.01) | | | 1.02 (0.9-1.04) |
| HMGB1^+^EVs/μL | 1.01(1.01-1.02) | | 1.02 (0.9-1.1) | | | 1.02 (0.9-1.1) |
| CRP,mg/L | 1.1 (1.02-1.3) | |  |  |  |  |
| IL-6,pg/L | 1.1 (1.01-1.2) | |  |  |  |  |
| VEGF, ng/L | 1.003(1.001-1.01) | |  |  |  |  |
| ICAM1,ng/mL | 1.1 (1.02-1.2) | |  |  |  |  |

The results are presented as OR (Odds ratio) with 95% CI (Confident interval).

aOR -adjusted OR for VEGF and IL6; aOR^1^ -adjusted OR for VEGF, IL6 and CRP.

Abbreviations: EEVs- endothelial extracellular vesicles; PEVs- platelet extracellular vesicles; LEVs- leucocytes extracellular vesicles; ICAM1^+^EVs- extracellular vesicles expressing Intercellular adhesion molecule 1; TF^+^EVs- extracellular vesicles expressing tissue factor; HMGB1^+^EVs extracellular vesicles expressing High mobility group box 1, SSc- systemic sclerosis; ILD- interstitial lung disease, CRP-C reactive protein; IL- interleukin; VEGF-Vascular Endothelial Growth Factor.
